# Taxonomic study of the genus *Thisizima* Walker, 1864 in China, with descriptions of two new species (Lepidoptera, Tineidae)

**DOI:** 10.3897/zookeys.254.3952

**Published:** 2012-12-21

**Authors:** Linlin Yang, Houhun Li, Roger C. Kendrick

**Affiliations:** 1 College of Life Sciences, Nankai University, Tianjin 300071, P. R. China; 2 Fauna Conservation Department, Kadoorie Farm & Botanic Garden, Lam Kam Road, Tai Po, New Territories, Hong Kong Special Administrative Region, P. R. China

**Keywords:** Lepidoptera, Tineidae, *Thisizima*, new species, China

## Abstract

The taxonomic study of the genus *Thisizima* Walker, 1864 is carried out in China. *Thisizima subceratella* **sp. n.** and *Thisizima fasciaria* **sp. n.** are described as new based on the specimens collected in Fujian, Hainan and Hong Kong. Detailed male and female genitalia are described for the first time for the genus. Photographs of adults and genital structures are provided. A checklist of all the described species is included.

## Introduction

The genus *Thisizima* was established by Walker (1864) with *Thisizima ceratella* Walker, 1864 as the type species. It includes five named species: *Thisizima ceratella* Walker, 1864 distributed in India, Burma, Thailand, West Malaysia and the Anambas Islands; *Thisizima antiphanes* Meyrick, 1894 in Burma and Thailand; *Thisizima sedilis* Meyrick, 1907 in Bhutan, Sikkim, Burma and Thailand; *Thisizima bubalopa* Meyrick, 1911 in Sri Lanka and India, and *Thisizima bovina* Meyrick, 1928 in the Andaman Islands (Robinson et al. 1994; Robinson 2008, 2009). Robinson(2009) further mentioned seven unnamed species occurring in India, the Andaman Islands, Thailand, Sarawak, Brunei and Hong Kong. Kendrick (2002) reported two *Thisizima* species occurring in Hong Kong in his PhD thesis: one was suspected to be *Thisizima ceratella* Walker, 1864, another was unnamed. However, no detailed description of the genitalia has been given for the genus except that Robinson et al. (1994) mentioned that the female has an invaginated corethrogyne. We herein describe the generic characters in detail, add two new species, *Thisizima subceratella* sp. n. and *Thisizima fasciaria* sp. n. to the genus, and provide a checklist of *Thisizima* on a worldwide basis.

## Material and methods

Specimens examined in this study were collected by light traps in Fujian, Hainan and Hong Kong. The type specimens are deposited in NKU and KFBG respectively.

Genitalia dissections were carried out following the methods described by Li (2002), and whole body dissections following methods described by Lee and Brown (2006). Photographs of the adults were taken with a Nikon D300 digital camera plus AF-S VR Micro-Nikkor 105mm f/2.8G IF-ED lens, and photographs of the genitalia were taken with an Olympus C7070WZ digital camera attached to an Olympus BX51 microscope.

### Abbreviations

**NKU** Insect Collection, College of Life Sciences, Nankai University, Tianjin, China.

**KFBG** Kadoorie Farm and Botanic Garden, Hong Kong, China.

**BMNH** Natural History Museum, London, UK.

**IMK** Indian Museum, Kolkata (Calcutta), India.

**HL** Holotype.

**PL** Paratype.

**ST** Syntype.

## Taxonomic accounts

### Thisizima

Walker, 1864

http://species-id.net/wiki/Thisizima

Thisizima Walker, 1864: 820.

#### Type species:

*Thisizima ceratella* Walker, 1864: 820, by monotypy.

#### Generic characters.

Head with tufts of erect piliform scales. Antenna (Figs 5, 6) about 1.2× length of forewing in male, and 0.75× length in female; scape expanded, pecten with fewer than 15 bristles; flagellum compressed broadly and flatly, although considerably broader in male, with appressed scales, cilia not visible without removal of scales. Maxillary palpus rather reduced, minute, 1/3 length of first segment of labial palpus, 4-segmented, segmental ratio 2:1:1.5:1. Labial palpus (Figs 7, 8) rather long, almost 2.5× height of head, curved, ascending; segmental ratio 1:2:2; second segment with dense forwards projecting piliform scales, with 6−7 lateral bristles; third segment with appressed scales, slender and pointed, without vom Rath’s organ. Forewing (Fig. 4) with costa moderately arched, apex roundly obtuse, termen obliquely rounded; unicolored or bicolored; all veins separate, R_1_ from basal 1/4 of cell, R_3_ from upper angle, R_5_ to apex, M_3_ close to CuA_1_ at base, CuA_1_ from lower angle of cell, forked portion of A_1+2_ about 1/3 length of vein, trace of CuP weak, cell closed, with trace of chorda and M stem; retinaculum in male subcostal, elongately triangular, with broad base and curled apex. Hind wing (Fig. 4) with costa moderately arched, apex rounded, slightly pointed than forewing, termen more oblique than forewing; all veins present, Rs parallel to Sc+R_1_, CuA_2_ from middle of lower margin of cell, trace of CuP weak, cell closed, with weak trace of M stem; frenulum with one stout bristle, sharp toward apex, angled at basal one third in male; also one bristle in female, much slender and shorter than in male. Legs smoothly scaled, except hind tibia with moderately elongate scales; foretibial epiphysis absent, hind tarsus without spine; tibial spur pattern 0-2-4, mid leg with outer spur about half length of inner spur, hind leg with outer mid spur about 0.4× length of inner spur, outer proximal spur about 0.6× length of inner spur.

Male genitalia. Corema absent in eighth segment. Vinculum rather broad, forming a strongly sclerotized cylinder with ill-defined tegumen. Uncus bilobate. Gnathos and subscaphium absent. Valva complex, base broad, scattered with strong hairs on outer surface; apodeme short, digitate. Transtilla absent.

Female genitalia. Corethrogyne present in seventh segment, invaginated into three intersegmental pouches. Ovipositor short and broad; papillae anales large, setose, sclerotized laterally; with one membranous dorsal sac and three ventral sacs between papillae anales, flower-like on top view. Apophyses short, apophysis posterior about 0.4× length of apophysis anterior. Ostium located on seventh sternite. Antrum distinct, anatropous.

#### Diagnosis.

The elongate, curved, ascending labial palpus of *Thisizima* is quite similar to that of *Tinissa* Walker, 1864 in Scardiinae, to that of *Euplocamus* Latreille, 1809 and *Psecadioides* Butler, 1881 in Euplocaminae. *Thisizima* can be distinguished by the antenna with flagellum strongly flattened in both sexes; while the antenna is filiform in *Tinissa*, and bipectinate in male and filiform in female in Euplocaminae. Besides, R_4_ and R_5_ on the forewing are separated in *Thisizima*, but stalked in the last three genera. The corethrogyne of *Thisizima* in female is invaginated into three intersegmental pouches that can also be found in Perissomasticinae, but its other female characters are quite different from those of the members in Perissomasticinae.

#### Biology.

Little is known about its biology. The living habitat is shown in figures 12 and 13.

#### Distribution.

China (Fujian, Hainan, Hong Kong); Burma, Thailand, West Malaysia, India, Sikkim, Bhutan, Sri Lanka, the Andaman Islands and the Anambas Islands.

#### Remarks.

*Thisizima* was placed in Tineidae since its establishment. The genus belongs to Tineidae without doubt, represented by the head with erect piliform scales, the subovate forewing with R_4_ terminating on costa, the male retinaculum arising from Sc, and the female abdomen with corethrogyne in the seventh segment. However, its subfamily position has not been assigned due to some characters that indicate the uniqueness of this genus: the strongly flattened antenna, the rather reduced maxillary palpus, the absence of foretibial epiphysis and the position of ostium. The shape of the labial palpus may suggest its affiliation with Scardiinae and Euplocaminae; the corethrogyne in female may suggest its affiliation with Perissomasticinae. The status of *Thisizima* might be settled with further work on its morphology and biology.

##### Checklist of the genus *Thisizima* Walker, 1864

**1. *Thisizima antiphanes* Meyrick, 1894**

*Thisizima antiphanes* Meyrick, 1894: 27.

Type locality: Burma.

Depository of type: BMNH (HT).

Distribution: Burma and Thailand.

**2. *Thisizima bovina* Meyrick, 1928**

*Thisizima bovina* Meyrick, 1928: 428.

Type locality: Andaman Islands.

Depository of type: Unknown.

Distribution: Andaman Islands.

**3. *Thisizima bubalopa* Meyrick, 1911**

*Thisizima bubalopa* Meyrick, 1911: 125.

Type locality: Sri Lanka (Peradeniya); India (Nilgiris).

Depository of types: BMNH (ST).

Distribution: Sri Lanka and India.

**4. *Thisizima ceratella* Walker, 1864**

*Thisizima ceratella* Walker, 1864: 820.

Type locality: India.

Depository of type: BMNH (HT).

Distribution: India, Burma, Thailand, West Malaysia and Anambas Islands.

**5. *Thisizima fasciaria* sp. n.**

Type locality: China (Fujian).

Depository of type: NKU (HT & PT), KFBG (PT).

Distribution: China (Fujian, Hong Kong).

**6. *Thisizima sedilis* Meyrick, 1907**

*Thisizima sedilis* Meyrick, 1907: 989.

Type locality: Bhutan; Sikkim.

Depository of type: IMK (ST).

Distribution: Bhutan, Sikkim, Burma and Thailand.

**7. *Thisizima subceratella* sp. n.**

Type locality: China (Fujian).

Depository of type: NKU (HT & PT), KFBG (PT).

Distribution: China (Fujian, Hainan, Hong Kong).

### Thisizima fasciaria sp. n.

urn:lsid:zoobank.org:act:A0755544-7CF8-4F5F-AE68-396A337FF41B

http://species-id.net/wiki/Thisizima_fasciaria

Figs 1, 2 10 −13

#### Type material.

**CHINA,** Holotype ♂, **Fujian Province:** Mt. Tianzhu (24°35'N, 117°55'E), Xiamen City, 220 m, 14.ix.2010, leg. Yinghui Sun & Jing Zhang (NKU).

**Paratypes:** 2 ♂, 15,19.viii.2010, leg. Bingbing Hu & Jing Zhang, same locality as holotype, genitalia slide No. YLL11172 (NKU). **Hong Kong:** 1 ♀, Kadoorie Agricultural Research Centre (22°25'N, 114°06'E), 210 m, 20.iv.2007, leg. Houhun Li *et al*. (NKU), genitalia slide No. YLL11165; 1 ♂, Kadoorie Agricultural Research Centre, 210 m, 20.ix.2009, leg. Houhun Li *et al*. (NKU); 1 ♂, Kadoorie Farm and Botanic Garden (22°25'N, 114°07'E), 315−575 m, 26.ix.2009, leg. Houhun Li *et al*. (NKU); 1 ♀, Kadoorie Agricultural Research Centre, Shek Kong, N.T., UTM: 50Q KV 030833, 28.iv.1997, 125W MBF, leg. R.C. Kendrick, genitalia slide No. YLL11171 (KFBG); 1 ♂, Kadoorie Agricultural Research Centre, Shek Kong, N.T., UTM: 50Q KK 029832, alt. 200 m, 6.v.1998, 125 W MBF, leg. R.C. Kendrick (KFBG); 1 ♂, Kadoorie Agricultural Research Centre, Shek Kong, N.T., UTM: 50Q KK 029832, alt. 200 m, 2.iv.1999, 125 W MBF, leg. R.C. Kendrick (KFBG).

#### Description.

**Figures 1−8.**
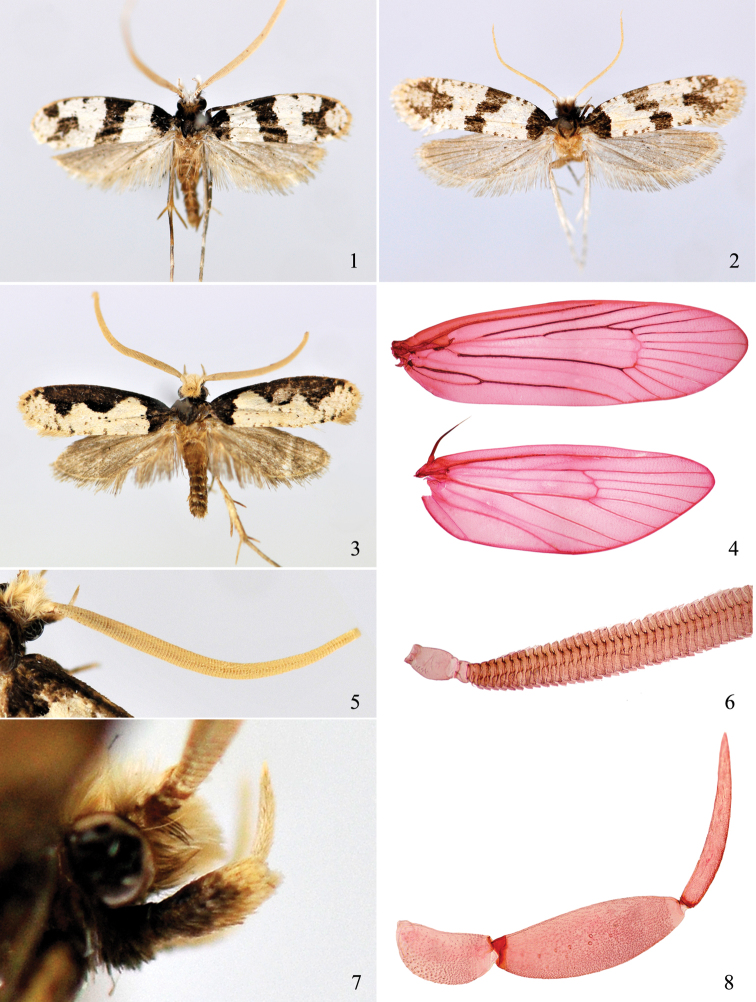
Morphology of *Thisizima* spp. **1** *Thisizima fasciaria* sp. n., holotype **2** *Thisizima fasciaria* sp. n., paratype **3** *Thisizima subceratella* sp. n., holotype **4−8** detailed structures of *Thisizima subceratella* sp. n., paratypes: **4** vein **5, 6** antenna **7, 8** labial palpus (**4, 6, 8** slide No. NKYLL010) (**1, 3−8** ♂, **2** ♀).

Imago (Figs 1, 2): Wingspan 13.0−15.0 mm in male, 17 mm in female. Vertex cupreous brown on posterior half, snow white on anterior half; frons snow white, with fine black scales on outer side before eyes. Antenna about 1.2× length of forewing in male, and 0.75× length in female including fringe; scape yellowish white above, brown mixed with black scales below, pecten with 10−15 black bristles; flagellum yellow, compressed broadly and flatly. Labial palpus snow white, first segment dark cupreous brown on outer surface, second segment cupreous brown on outer surface of basal half, with sparse black lateral bristles. Thorax and tegula black. Forewing index about 0.32; ground color bright white; a black triangular patch from costal margin to dorsum on basal 1/6; an oblique, black fascia from basal 1/3 to just before middle of dorsum, slightly narrowed medially, sinuate along both margins; a rectangular black patch from outer margin of cell to distal 1/6 of forewing, confluent with two black subtriangular patches from costa and termen before apex respectively, forming a broad Y-shaped pattern; two black costal spots between oblique fascia and Y-shaped pattern; termen and dorsum scattered with faint dark brown dots, dim in some specimens; fringe yellowish brown. Hindwing index 0.35; light grayish brown; fringe gray; frenulum with one stout bristle in male, one much slender and shorter bristle in female. Fore leg black; mid leg black, with snow white fine scales at apex of tibia, tarsus yellowish brown on ventral surface, with white at apex of each segment on dorsal surface, spurs dark brown; hind leg and spurs yellowish brown, apex of tibia and each segment of tarsus with white scales dorsally.

**Male genitalia** (Fig. 10). Vinculum convex anteriorly in arch, slightly convex at middle on posterior margin. Uncus sclerotized, trapezoidal, semicircularly concave at middle on posterior margin; uncus lobes short, setose. Valva rounded in basal half, horn-shaped and curved outwardly in distal half, setose on outer surface. Juxta small, subovate. Aedeagus slightly curved ventrad, with a pair of blade-shaped lateral sclerites connected by membrane dorsally and opened ventrally, apex sharp obliquely; cornutus absent.

**Female genitalia** (Fig. 11). Seventh tergite short, membranous except heavily sclerotized anteriorly; sternite slightly sclerotized, 0.2× as long as broad, tapered posteriorly, produced to a slender, elongate plate at middle. Eighth tergite rectangular, with dense, minute spinules, with short spines along posterior margin; sternite rectangular, with dome-like membranous section medially, scattered with short spinules, sclerotized section with dense short spinules. Ostium at anterior 1/3 of seventh sternite, rounded. Antrum (Fig. 11a) heavily sclerotized, funnel-shaped, with a more sclerotized pouch anteriorly on ventral surface. Ductus bursae about 1.4 × length of corpus bursae, posterior 1/6 slender, anterior 5/6 broadened; inception of ductus seminalis at posterior 1/6 of ductus bursae. Corpus bursae elongate oval, with a broad ring-shaped, punctate signum at posterior 1/3 (Fig. 11b).

**Figures 9–11.**
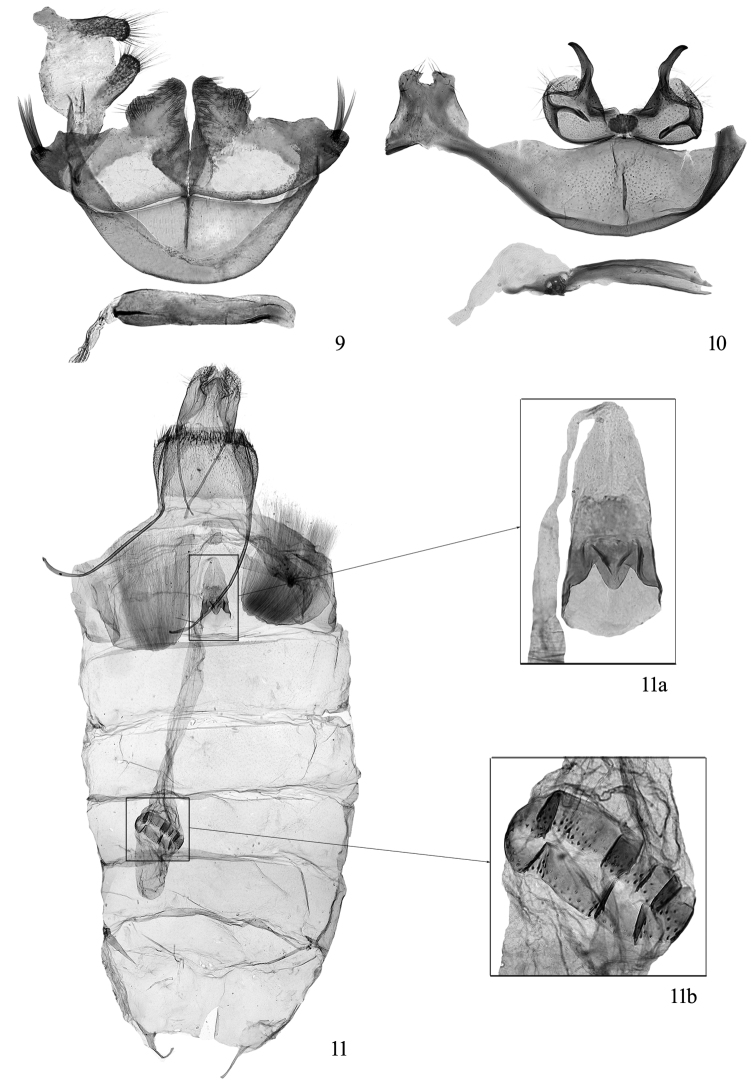
Genitalia of *Thisizima* spp. **9** male genitalia of *Thisizima subceratella* sp. n., paratype, slide No. NKYLL010 **10−11** *Thisizima fasciaria*sp. n., paratypes: **10** male genitalia, slide No. YLL11172 **11** female genitalia (11a. antrum; 11b. signum), slide No. YLL11156.

#### Diagnosis.

The bold, bicolored forewing pattern of the new species is diagnostic: ground color bright white with a triangular black patch at base, an oblique, black fascia near middle and a somewhat Y-shaped black patch near apex. There are four other species with bicolored forewing pattern: *Thisizima antiphanes* has a white basal band and an apical dot, *Thisizima sedilis* has a large rounded-triangular costal blotch, *Thisizima ceratella* has a broad irregular costal stripe broadly confluent with basal patch on anterior margin, and *Thisizima subceratella* sp. n.has a broad black costal blotch narrowly confluent with basal patch on anterior margin.

#### Biology.

**Figures 12–13.**
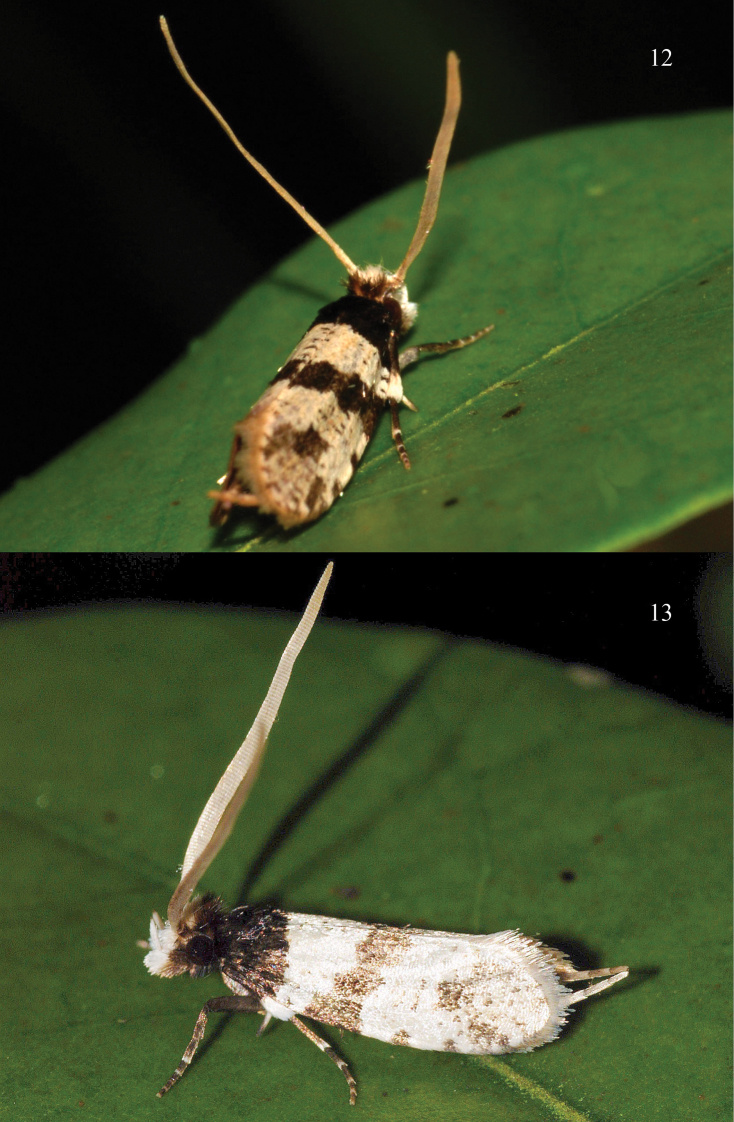
Adults of *Thisizima fasciaria* sp. n. in repose. **12** Shan Liu, Sai Kung, Hong Kong, 9.iv.2010, photographed by R.C. Kendrick **13** Hong Kong, photographed by R.C. Kendrick.

The living habitat is shown in figures 12 and 13.

#### Distribution.

China (Fujian, Hong Kong).

#### Etymology.

This specific name is derived from the Latin *fasciarius*, meaning fascia, referring to the oblique, black fascia near middle of forewing.

### Thisizima subceratella sp. n.

urn:lsid:zoobank.org:act:FA0F81DD-9A18-4261-BB92-F89F9681B288

http://species-id.net/wiki/Thisizima_subceratella

Figs 3−9

#### Type material.

**CHINA,** Holotype ♂, **Fujian Province**: Mt. Tianzhu (24°35'N, 117°55'E), Xiamen City, 220 m, 12.ix.2010, leg. Yinghui Sun & Jing Zhang (NKU).

**Paratypes:** 2 ♂, 30.viii,19.ix.2010, other same data as holotype, genitalia slide No. NKYLL010 (NKU). **Hainan Province:** 1 ♂, Mt. Wuzhi (18°31'E, 109°24'E), 700 m, 19.v.2007, leg. Zhiwei Zhang & Weichun Li (NKU). **Hong Kong:** 2 ♂, Kadoorie Agricultural Research Centre (22°25'N, 114°06'E), Shek Kong, N.T., UTM: 50Q KV 030833, 125 W MBF, 28.iv.1997, leg. R.C. Kendrick (KFBG).

#### Description.

Imago (Figs 3−8). Wingspan 18.5−20.5 mm in male. Vertex and frons yellowish white, tinged with brown around eyes. Antenna about 1.2× length of forewing including fringe; scape yellowish white above, scattered with brown scales below, pecten with 8−12 black bristles; flagellum ochreous yellow, compressed broadly and flatly. Labial palpus yellowish white, first segment black on outer surface, second segment black on outer surface of basal 2/3, with sparse black lateral bristles. Thorax black, tegula black on anterior half and dark cupreous brown on posterior half. Forewing index about 0.3; ground color creamy white, with scattered dark brown scales on distal 1/4; a triangular black patch on basal 1/5, broader at costa, sinuate along outer margin; a broad black costal blotch narrowly confluent with basal patch on anterior margin, curved in W shape on posterior margin, its basal half apically rounded and reaching half width, distal half triangularly crossing midwing; two small indistinct spots along costa before apex; termen and dorsum scattered with faint dark brown dots; fringe light yellowish brown. Hind wing index 0.38; cupreous brown; fringe grayish brown; frenulum one stout bristle. Fore leg black except distal half of coxa yellowish brown on ventral surface; mid leg black except tarsus yellowish brown on ventral surface, spurs dark brown; hind leg yellowish brown mixed with black scales.

**Male genitalia** (Fig. 9). Vinculum convex anteriorly in arch, slightly convex at middle on posterior margin; with a longitudinal suture in middle posteriorly, 0.5× length of vinculum. Uncus membranous, trapezoidal; uncus lobes digitate, slightly sclerotized, with elongate setae on ventral surface. Valva with base broad and gently arched, connected ventrally at base by a sclerotized, somewhat funnel-shaped plate on inner surface; basal 2/3 membranous and inverted fan-shaped, scattered with strong hairs on outer surface; apex as wide as base, heavily sclerotized, sinuate, dorsoapically with a tuft of 3−4 strong spines, ventroapically convex, with dense, stout spines on outer surface. Juxta membranous. Aedeagus membranous dorsally, sclerotized ventrally, with a shallow keel at base; cornutus absent.

**Female.** Unknown.

#### Diagnosis.

The new species is quite similar to *Thisizima ceratella*, but can be separated by the forewing having a broad blackish costal blotch narrowly confluent with the basal patch on anterior margin, curved in W-shape on posterior margin; while *Thisizima ceratella* has an irregular cupreous black costal stripe broadly confluent with the basal patch on anterior margin, curved outward before middle on posterior margin.

#### Distribution.

China (Fujian, Hainan, Hong Kong).

#### Etymology.

The specific name is derived from the Latin prefix *sub*-, meaning similar, and another specific name *ceratella*, referring to the similarities of the two species.

#### Remarks.

K. Tuck (BMNH) assisted us to check the identity of *Thisizima ceratella*. Unfortunately, the holotype has lost its hindwings and abdomen. The late G. Robinson had therefore dissected a male specimen identified as *ceratella* in the Meyrick collection, collected in Koni, Burma. Tuck kindly compared our illustrations of *Thisizima subceratella* sp. n.with Robinson’s slide BMNH Microlep. No. 27736. He noticed a small but distinct difference in the shape of the valva: in Robinson’s dissection the valva is slightly larger and therefore extends further laterally and has five strong spines, whereas our illustration shows a relatively short valva with only 3−4 spines on each.

Furthermore, the adult photograph of *Thisizima ceratella* given by Robinson et al. (1994) shows that the forewing pattern is coincident with Walker’s original description. We thus base our understanding of the identity of *Thisizima ceratella* on this dissected specimen.

There are many tineid species showing small differences in genitalia, but they can usually be recognized by the external morphology, such as forewing pattern and venation (eg. species of *Monopis* Hübner). The forewing pattern in the new species is quite different from that of *Thisizima ceratella*, and the shape of the valva does have small but distinct difference between the two species, which we regard as sufficient evidence that this is a good species.

## Supplementary Material

XML Treatment for Thisizima

XML Treatment for Thisizima fasciaria

XML Treatment for Thisizima subceratella
